# Construction of a Chimeric Secretory IgA and Its Neutralization Activity against Avian Influenza Virus H5N1

**DOI:** 10.1155/2014/394127

**Published:** 2014-02-13

**Authors:** Cun Li, Xiaoping An, Azeem Mehmood Butt, Baozhong Zhang, Zhiyi Zhang, Xiaona Wang, Yong Huang, Wenhui Zhang, Bo Zhang, Zhiqiang Mi, Yigang Tong

**Affiliations:** ^1^State Key Laboratory of Pathogen and Biosecurity, Beijing Institute of Microbiology and Epidemiology, Beijing 100071, China; ^2^Centre of Excellence in Molecular Biology (CEMB), University of the Punjab, Lahore-53700, Pakistan

## Abstract

Secretory immunoglobulin A (SIgA) acts as the first line of defense against respiratory pathogens. In this assay, the variable regions of heavy chain (VH) and Light chain (VL) genes from a mouse monoclonal antibody against H5N1 were cloned and fused with human IgA constant regions. The full-length chimeric light and heavy chains were inserted into a eukaryotic expressing vector and then transfected into CHO/dhfr-cells. The chimeric monomeric IgA antibody expression was confirmed by using ELISA, SDS-PAGE, and Western blot. In order to obtain a dimeric secretory IgA, another two expressing plasmids, namely, pcDNA4/His A-IgJ and pcDNA4/His A-SC, were cotransfected into the CHO/dhfr-cells. The expression of dimeric SIgA was confirmed by using ELISA assay and native gel electrophoresis. In microneutralization assay on 96-well immunoplate, the chimeric SIgA showed neutralization activity against H5N1 virus on MDCK cells and the titer was determined to be 1 : 64. On preadministrating intranasally, the chimeric SIgA could prevent mice from lethal attack by using A/Vietnam/1194/04 H5N1 with a survival rate of 80%. So we concluded that the constructed recombinant chimeric SIgA has a neutralization capability targeting avian influenza virus H5N1 infection in vitro and in vivo.

## 1. Introduction

Endemic highly pathogenic avian influenza virus (AIV) H5N1 in poultry has been present since the first occurrence in 1997 in Hong Kong. AIV H5N1 circulates in waterfowl and domesticated avian species and has evolved into multiple phylogenetically distinct genotypes and clades [1–3], with geographically distinct groups in each country. H5N1 viruses occasionally infect humans, with high case-fatality rates. These viruses have repeatedly crossed the species barrier and caused highly lethal human infections. The wide distribution of highly pathogenic AIV H5N1 is a global threat to human health [4–7]. Most deaths have occurred in young, previously healthy, adults or children. According to the latest WHO report [8], there have been 633 laboratory-confirmed highly pathogenic H5N1 AI cases worldwide from 2003 to 2013, with a mortality of 59.6%.

For active immunization, vaccination would be ideal; however, there are some problems with avian influenza (AI) vaccines at present. There is no current pandemic of AI in humans, and therefore it is difficult to accurately assess the protective effects of any vaccine. Vaccines also have a major drawback because it would take several weeks to produce protective antibodies. This often reduces preventative effects and obstructs their effectiveness as emergency protection, especially in some high-risk groups. In contrast, passive immune agents can make up for the deficiencies of vaccines and can generate protective effects immediately after administration.

Research into passive immunity for AI prevention and treatment has been intensive in recent years. Animal experiments have shown that either polyclonal (serum, plasma) [9–11] or monoclonal [12–16] antibodies offer good protection against highly pathogenic AI. Meanwhile, many researchers have reported antibodies providing broad cross-protection against AIV H5N1 [12, 14, 17–19].

As a respiratory disease, AIV infection occurs via respiratory or digestive tract mucosa. Secretory IgA (SIgA), first identified in the 1960s, is a type of IgA antibody found in breast milk, gastrointestinal fluids, respiratory secretions, and genitourinary tracts. SIgA consists of two monomeric IgA units, which are associated with the J chain acquired during the process of polymerization in plasma cells just before secretion, along with the secretory component (SC) [20, 21]. SIgA is considered the first-line defense in mucosal immunity and plays a critical role in preventing pathogen adhesion to host cells, thereby blocking dissemination and further infection. Because of its dimeric structure, SIgA has a higher functional affinity [22]. In vitro, SIgA is more resistant to proteases than serum IgA [23–25]. Its half-life is three times longer than IgG on mucosal surfaces, and it can provide a specific protective effect for at least 4 months [22]. The presence of the SC also gives SIgA special protective immunity activity. First, the SC has nonspecific activity against pathogenic microorganisms [26]. Second, via carbohydrate residues, SIgA can adhere to epithelial surfaces, forming a protective layer and effectively preventing invasion by a virus [27, 28]. It would be of great significance to demonstrate the blocking effects of SIgA against AIV infection in the respiratory or digestive tracts.

Previous reports have shown that IgA can potentially be used for passive protection or therapeutic intervention on mucosal surfaces. IgA can act as a neutralizing antibody against pathogens and exotoxins, with better affinity than neutralizing antibodies of other classes [29]. Monoclonal IgA antibodies against respiratory syncytial virus were applied passively to the nasopharyngeal mucosa and prevented subsequent infection and pneumonia [30]. Passive oral delivery of IgA antibodies also protected against bacterial infections in the intestine of mice [31]. IgA has lower proteolytic stability without the bound SC [23, 24], and therefore it may be efficient to use purified SIgA as a passive treatment agent. In this study, we constructed a mouse and human derived SIgA and explored its feasibility in preventing H5N1 virus infection. Our results revealed that the recombinant SIgA could act as a preventative agent against H5N1 infection.

## 2. Materials and Methods

### 2.1. Reagents, Cells, and Virus

Restriction endonucleases and T4 Ligase for cloning were obtained from New England Biolabs (Beverly, MA, USA). Lipofectamine 2000 was purchased from Gibco BRL (Gaithersburg, MD, USA), with protein A-agarose, plasmid pcDNA4/His A, and zeocin from Invitrogen (Carlsbad, CA, USA). Dulbecco's Modified Eagle's Medium (DMEM) and Fetal Bovine Serum (FBS) were obtained from Hyclone (Logan, UT, USA). A hybridoma cell line secreting an anti-H5N1 (HA) antibody HA-9 was a gift from Dr. Chen (Beijing Institute of Microbiology and Epidemiology). A One-Step RT-PCR Kit was purchased from Takara (Dalian, China). The eukaryotic expression vector pEF-dhfr2a-NEO [32] was constructed in our laboratory. The CHO cells were cultured in DMEM containing 10% fetal calf serum. Madin-Darby canine kidney (MDCK) cells were purchased from the American Type Culture Collection (ATCC, Manassas, VA). Cells were used for a maximum of 25 passages and maintained in DMEM containing 5% FBS, 2 mM L-glutamine, penicillin, and streptomycin (Gibco BRL). Cultures were incubated at 37°C/5% CO_2_ in a humidified atmosphere. Aliquots of H5N1 influenza A virus A/Vietnam/1194/04 grown in embryonated eggs was stored at −70°C. The 50% tissue culture infectious dose (TCID_50_) was determined by titration in MDCK cells.

### 2.2. Cloning of the HA-9 Variable Region and Construction of the Vector Expressing Heavy Chain

Total RNA was extracted from hybridoma cells using Trizol (Invitrogen), and the variable region gene was cloned by using a Takara One-Step RT-PCR Kit according to the manufacturer's instructions. The variable region of the HA-9 light chain (mVL) gene was cloned with primers mVK-f-ATG and mVK-r. The heavy chain variable gene (mVH) was cloned using primers mVH-f-ATG, and mVH-r. PCR products were recovered and inserted into pMD18-T, then verified by sequencing. Sequences of the primers used in this assay were shown in Table 1.

Plasmid pGEM-T-Easy-IGHA [33] was digested with *EcoRI*. The 1400 bp fragment carrying the IgA2 heavy chain constant region gene (IGHA2) was recovered. mVH was recovered from pMD18-T-mVH by digesting with *EcoRI* and *SalI*. A mixture of IGHA2 and mVH was amplified by overlap PCR with primers IGA-mVHhCHf, IGA-mVHhCHr, IGA-Hf-*EcoRI*, and IGA-Hr-*XbaI*. Products of the primary PCR were used as the template for amplifying the full-length chimeric IgA in the secondary PCR with primers IGA-Hf-*EcoRI* and IGA-Hr-*XbaI*. The PCR products were recovered and then cloned into pMD18-T. The resulting recombinant plasmid was designated pT-CHI-mVH-IGHA, then verified by sequence analysis. A fragment (1.80 kb) was recovered by digesting pT-CHI-mVH-IGHA with *EcoRI* and *XbaI*. This fragment was then ligated into *EcoRI*/*XbaI*-treated pEF-dhfr2a-NEO, creating the expression vector pEF-dhfr2a-NEO-IGA-chi-H.

### 2.3. Construction of the Vector Expressing Light Chain

pGEM-T-Easy-CK was digested with *EcoRI*, and a 400 bp fragment encoding the light chain constant region (CK) was recovered. mVK was recovered from pMD18-T-mVK by digesting with *EcoRI* and *SalI*. The mixture of CK and mVK was amplified by overlap PCR with primers IGA-mVKhCKf, IGA-mVKhCKr, IGA-Kf-*EcoRI*, and IGA-Kr-*XbaI*. Products of the primary PCR were used as the template to amplify the full-length light chain of chimeric IgA in the secondary PCR (IGA-Kf-*EcoRI* and IGA-Kr-*XbaI*). PCR products were recovered and cloned into pMD18-T to yield pT-CHI-mVK-IGK, which was verified by sequence analysis. A 700 bp fragment was recovered by digesting pT-CHI-mVK-IGK with *EcoRI* and *XbaI*. The fragment comprising the light chain DNA was then ligated into *EcoRI*/*XbaI*-treated pEF-dhfr2a-NEO, creating expression vector pEF-dhfr2a-NEO-IGA-Kappa.

### 2.4. Construction of the Vectors Expressing SC or J Chain

**Figure 1:**
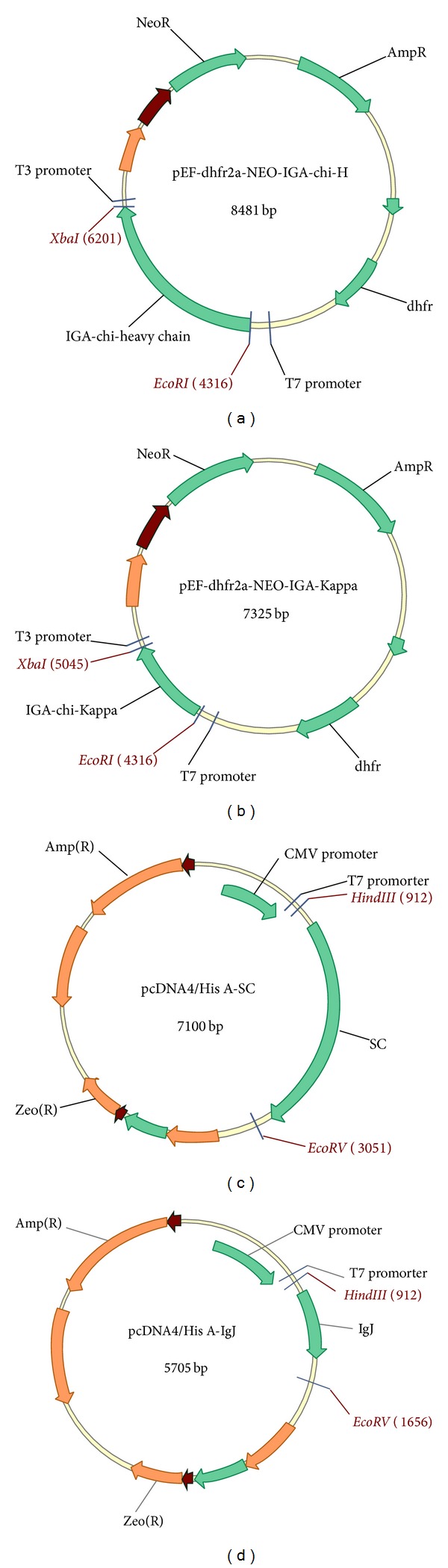
Schematic of the expression vectors. pEF-dhfr2a-NEO-IGA-chi-H and pEF-dhfr2a-NEO-IGA-Kappa contain the marker neoR and dhfr-. pcDNA4/His A-SC and pcDNA4/His A-IgJ contain the ORF for zeocin resistance (zeoR). The restriction sites used for the construction of the vectors are indicated.

According to previously published techniques [33, 34], we obtained the full-length pIgR gene and pcDNA4/His A-pIgR [33]. The human SC is composed of the first 585 amino acid residues of the polymeric Ig receptor. Using a published protocol, the “DREAM” technique [35], we mutated the intracellular region of pIgR, thereby generating pcDNA4/His A-SC expression vector. Using the same technique [33], we acquired the IgJ gene, ligated it into the expression vector pcDNA4/His A, and produced the expression plasmid, pcDNA4/His A-IgJ. All the above recombinant vectors were illustrated in diagram (Figure 1).

### 2.5. Establishment of IgA- and SIgA-Expressing CHO Cell Line

The CHO dhfr-cells were cultured in DMEM supplemented with 10% FBS, 100 *μ*M hypoxanthine, and 16 *μ*M thymidine. The cell concentration was adjusted to 2 × 10^5^ cells/mL by diluting with DMEM containing 10% FBS and seeded into six-well plates. Lipofectamine 2000 (4 *μ*L) was mixed with pEF-dhfr2a-NEO-IGA-chi-H (2 *μ*g) and pEF-dhfr2a-NEO-IGA-Kappa (2 *μ*g), respectively, in a final volume of 200 *μ*L of DMEM. The mixture was incubated for a further 20 min then gently added to one well of the six-well plate. After a 6 h incubation at 37°C/5% CO_2_, cells were fed with DMEM lacking hypoxanthine and thymidine, but containing 10% dialyzed FBS. Cells stably secreting IgA were screened in 96-well culture dishes, with selection continuing for 3 weeks. Clones expressing IgA were screened by using ELISA. A cell clone, which produced the most IgA, was amplified to serve as the recipient cell for subsequent transfections with pcDNA4/His A-SC and pcDNA4/His A-IgJ. Selection was carried out in the presence of 500 mg/mL Zeocin over 3 weeks. Cells producing SIgA were then amplified to be selected in methotrexate (MTX), the concentration of which was gradually increased.

### 2.6. Detection of Produced IgA and Determination of Its Antigen Binding Capacity

Sandwich ELISA was conducted to detect the production of IgA. A mouse anti-human IgA *α*-chain-specific monoclonal antibody (Sigma-Aldrich, diluted 1 : 3000 in 0.05 M Na_2_CO_3_ buffer, pH 9.6) was used to coat the wells (50 *μ*L/well) of Costar immunoplates overnight at 4°C. The plates were blocked with 5% skim milk in PBS followed by washing three times with PBS containing 0.05% Tween 20 (PBST). IgA supernatant (50 *μ*L) and human IgA (saliva) were then added to the wells and incubated for 2 h at 37°C. After extensive washing with PBST, HRP-conjugated goat anti-human IgA (InvivoGen) diluted 1 : 2500 in 2% skim milk in PBST was added (50 *μ*L/well). After incubation at 37°C for 1 h, further washes were followed by the addition of 3,3′,5,5′-tetramethylbenzidine for 15 min at 37°C. The assay was stopped with the addition of 50 *μ*L of 2 M H_2_SO_4_ and the absorbance at 450 nm measured immediately. The antigen binding capacity of the antibodies was determined by using a sandwich ELISA as described above. The capture antigen for this ELISA was A/Vietnam/1194/04 H5N1 HA antigen (500 ng/well), purified from the lysate of virus. The Bovine Serum Albumin (BSA) was coated on the wells as negative control.

### 2.7. SIgA Western Blot Analysis

CHO culture supernatant (1 mL) was incubated with rabbit anti-human *α*-chain-specific antibody (30 *μ*L, Sigma-Aldrich) overnight at 4°C. Protein-A sepharose (50 *μ*L, Sigma-Aldrich) equilibrated in PBS was added and the mixture incubated for 4 h at 4°C then centrifuged (3000 ×g, 4°C, 1 min). The resulting pellet was washed three times with 1 mL of PBS. The immunoprecipitated protein was separated by reduced or nonreduced SDS-PAGE, and then transferred to polyvinylidene difluoride membranes. Membranes were blocked overnight at 4°C by incubating with 10% skim milk in PBST, then incubated for 2 h at 37°C with the following primary antibodies, respectively: mouse anti-human IgA, *α*-chain specific (1 : 3000; Abcam, Cambridge, MA, USA); mouse anti-human, *κ*-chain specific (1 : 3000; Sigma Aldrich); mouse anti-human, J chain specific (1 : 2500; Abcam); and mouse anti-human, SC specific (1 : 3000; Sigma Aldrich). Bound antibodies were detected with an HRP-conjugated goat anti-mouse IgG (1 : 3000; Sigma Aldrich) in combination with enhanced chemiluminescent reagents (Millipore, Bedford, MA, USA).

### 2.8. SIgA Fermentation and Purification

Monoclonal SIgA cells were adapted to serum-free medium CD CHO medium (Gibco, Carlsbad, CA, USA) with 125 nM MTX over 2 months. The concentration of DMEM and dialysis serum was gradually reduced until cells were fully adapted to CD CHO medium. The adapted cell lines were grown in suspension cultures for 2–4 weeks in CD CHO medium containing 125 nM MTX. Culture supernatant was collected and purified using Pierce Protein L Chromatography Cartridges (Thermo Scientific, Hudson, NH, USA). After centrifugation (5000 ×g, 10 min), the supernatant was passed through a 0.45 *μ*m filter. The filtrate was then loaded onto a Protein L affinity column and the bound antibody was eluted with buffer (0.2 M Na_2_HPO_4_, 0.1 M citric acid pH 3.0). The antibodies were concentrated using a Centriplus YM-100 ultrafiltration tube (Pierce Chromatography Cartridges Protein-L) and the buffer replaced with sterile PBS. The resulting solution was aliquoted and stored at −20°C until required.

### 2.9. Neutralization Assay and Statistical Analysis

All microneutralization assays were performed on MDCK cells, with cells used for a maximum of 25 passages. The virus TCID_50_ was also determined by titration on these cells. Briefly, serial 10-fold dilutions of virus were made in DMEM containing 1% BSA, and a 100 *μ*L of each dilution was dispensed into 96-well plate. Freshly trypsinized MDCK cells were adjusted to a concentration of 1.5 × 10^5^ cells/mL and aliquots (100 *μ*L) added to each well. Each dilution was assayed in triplicate. Plates were covered and incubated at 37°C/5% CO_2_. Wells where a cytopathic effect (CPE) was observed were considered to be positive for virus growth. The TCID_50_ was calculated by the method of Reed and Muench [36].

Purified recombinant SIgA (10 mg/mL) was serially diluted 2-fold, with 50 *μ*L of each dilution added, in triplicate, to wells of a 96-well plate. Virus (100 *μ*L) containing 100 TCID_50_ was added to each well. Freshly trypsinized MDCK cells were adjusted to a concentration of 1.5 × 10^5^ cells/mL and aliquots (100 *μ*L) added to each well. The reciprocal of the last dilution at which infection was completely blocked was determined as the microneutralization titer of the virus stock.

All animal studies were approved by the Institutional Animal Care and Use Committee at Beijing Institute of Microbiology and Epidemiology, Beijing, China, and performed according to institutional guidelines for animal welfare. Mice were housed with 8 per cage and maintained on a 12 hour light/ dark cycle (lights on at 7:00 am) with continuous access to food and water. At the end of animal study mice were sacrificed by CO_2_ asphyxiation in accordance with the guidelines. Female BALB/c mice (6 weeks old) were kept in biosafety level 3 housing. All experimental protocols followed the standard operating procedures of the biosafety level 3 animal facilities. Aliquots of influenza A H5N1 A/Vietnam/1194/04 stocks were grown in embryonated eggs. Virus-containing allantoic fluid was harvested and stored in aliquots at −70°C. The LD_50_ was determined in mice following serial dilution of the virus stock. For the viral challenge, 10 LD_50_ were used in all experiments. Infection was established by intranasal inoculation of mice anesthetized with ketamine. The mice of negative control group (SIgA) were administrated with 20 *μ*L of saline and 20 *μ*L of SIgA (10 mg/mL), and those of positive control group (Challenge) were given 20 *μ*L of saline and 20 *μ*L of virus (10 LD_50_). Mice in Group 1 (SIgA + Challenge) were first administrated with 20 *μ*L of SIgA, and then 20 *μ*L of virus (10 LD_50_) 2 h later. Mice in group 2 (Challenge + SIgA) were first administrated with 20 *μ*L of virus (10 LD_50_), and then 20 *μ*L of SIgA 2 h later. Each group contains 8 mice.

Statistical analysis of the SIgA binding capacity data was performed by using an independent *t*-test. Survival data was analyzed by using a Kaplan-Meier survival analysis with a log-rank method of statistics. *P* < 0.05 was considered significant.

## 3. Results

### 3.1. Construction of SIgA Expression Vectors

The variable regions of the heavy (VH) and light (VL) chain genes of the anti-H5N1 HA neutralizing monoclonal antibody were cloned by RT-PCR [37]. These genes were analyzed by using the England Bioinformatics Institute website (IMGT/V-QUEST). The signal peptides for VH and VL were predicted by using artificial neural networks and hidden Markov models on the Danish Centre for Biological Sequence Analysis website (http://www.cbs.dtu.dk/services/SignalP/). The results indicated that VH and VL were derived from mouse immunoglobulin genes. The VH comprised 414 bp, with the first 57 nucleotides encoding a signal sequence. The genes for VH, D, and J were originally derived from mouse antibody genes IgHV I, IgHV IV, and IgHV II, respectively. The VL contains 393 bp, with the first 60 nucleotides encoding a signal sequence. The V region and J genes were derived from mouse antibody genes IgKV III and IgKJ I, respectively. To improve production of IgA in eukaryotic cells, the cloned IGHA sequence contained all introns [38].

### 3.2. Screening and Antigen Binding of SIgA

**Figure 2:**
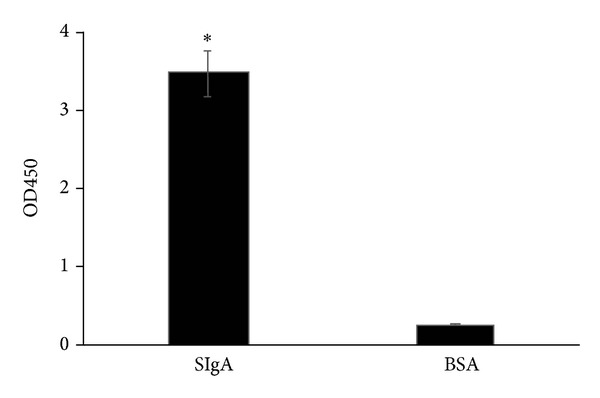
ELISA analysis of H5N1 HA antigen binding capacity of SIgA. H5N1 HA or BSA was coated on 96-well plate as the capture antigens. The produced SIgA was added as the primary antibody, and the secondary antibody was HRP-conjugated goat anti-human IgA. Data were the average of three independent experiments and shown as mean ± SD. Significance was determined by using independent *t*-test (*P < 0.05).

Sandwich ELISA was used to screen the cell clones expressing IgA and SIgA (data not shown), and the clone 6 which produces the most amount of SIgA was selected in the following assays. The capacity of the produced SIgA binding to antigens was evaluated by using the Sandwich ELISA, and the H5N1 HA was used as a capturing antigen. The results showed that the produced recombinant SIgA could bind to the H5N1 HA antigen (Figure 2).

### 3.3. SIgA-Specific Western Blot Analysis

**Figure 3:**
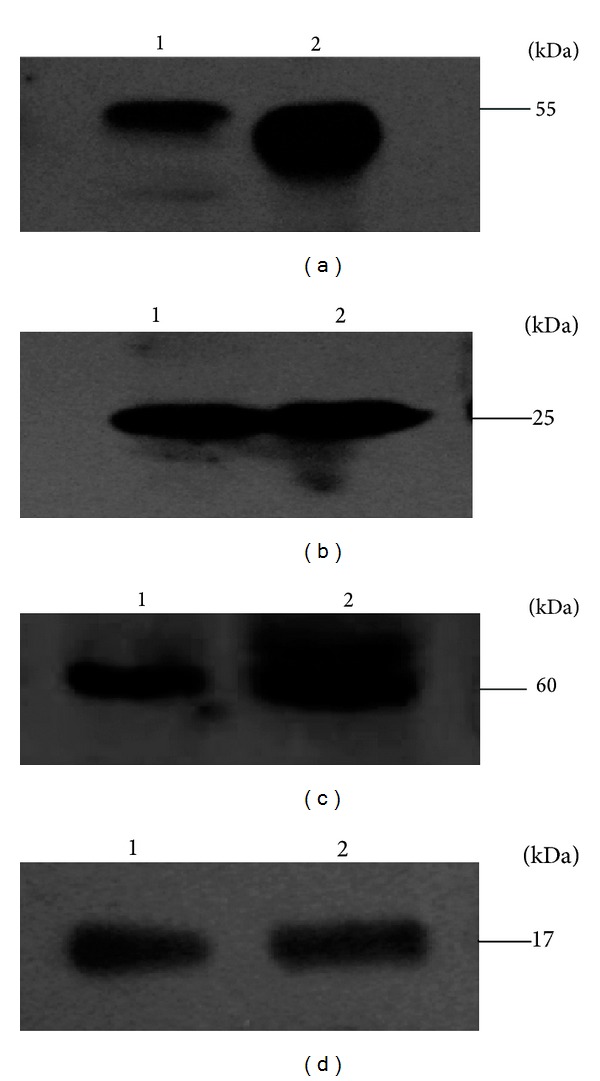
Western blot analysis of recombinant SIgA under reduced conditions. Transferred proteins were incubated with various specific primary antibodies. (a) Mouse anti-human IgA *α*-chain. (b) Mouse anti-human *κ*-chain. (c) Mouse anti-human SC. (d) Mouse anti-human J chain. HRP-conjugated goat anti-mouse IgG was used as secondary antibody. Lane 1: human saliva (positive control); 2: produced SIgA.

**Figure 4:**
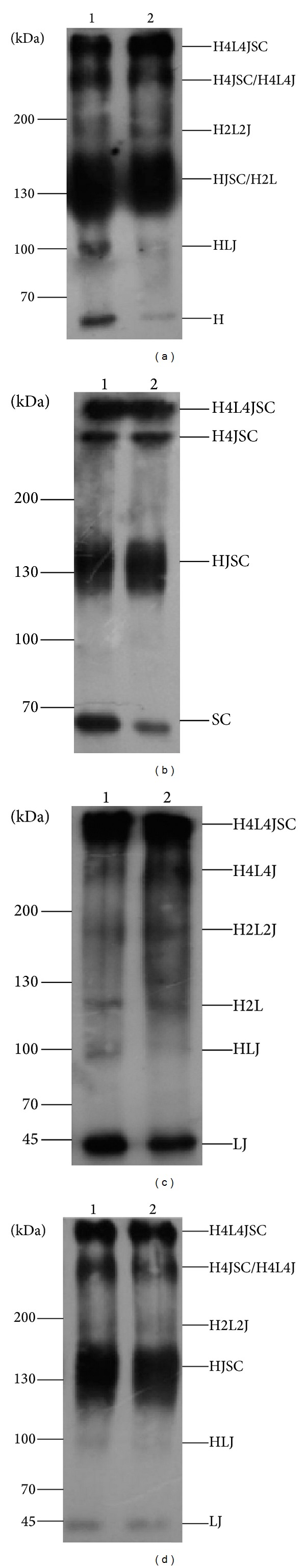
Western blot analysis of recombinant SIgA under nonreducing conditions. Transferred proteins were incubated with different primary antibodies to confirm the various polymeric forms of recombinant SIgA. (a) Mouse anti-human IgA *α*-chain antibody. (b) Mouse anti-human SC antibody. (c) Mouse anti-human *κ*-chain antibody. (d) Mouse anti-human J chain antibody. HRP-conjugated goat anti-mouse IgG was used as secondary antibody. Lane 1: produced SIgA; 2: human saliva (positive control). H4L4JSC (complete SIgA molecule), H4JSC/H4L4J (four IgA *α*-chains, J chain, and secretory component or four IgA *α*-chains, four *κ*-chains, and J chain), HJSC/H2L (one IgA *α*-chain, J chain, and secretory component or two IgA *α*-chains and one *κ*-chain), HLJ (one IgA *α*-chain, one *κ*-chain, and J chain), LJ (one *κ*-chain and J chain), and H or SC (monomer of IgA *α*-chain or secretory component).

Recombinant chimeric SIgA produced by cells was detected by western-blotting under reduced and non-reduced conditions. The Recombinant chimeric SIgA was shown to consist of four subunits: an IgA *α* chain (55 kDa); a kappa light chain (25 kDa); a J chain (17 kDa); and the SC (66 kDa). These four subunits were similar to those observed from human saliva SIgA (Figure 3). Results of SDS-PAGE under nonreducing conditions demonstrated that the recombinant SIgA presented as different composed patterns and was exactly the same as naturally-secreting SIgA (Figure 4). The band of H4L4JSC is the complete SIgA molecule. The band of H4JSC/H4L4J is composed of four IgA *α*-chains, J chain and secretory component or of four IgA *α*-chains, four *κ*-chains, and J chain. The band of HJSC/H2L is composed of one IgA *α*-chain, J chain, and secretory component or of two IgA *α*-chains and one *κ*-chain. The band of HLJ is composed of one IgA *α*-chain, one *κ*-chain and J chain. The band of LJ is composed of one *κ*-chain, and J chain. The band of H or SC means the monomer of IgA *α*-chain or secretory component.

### 3.4. Purification of SIgA

**Figure 5:**
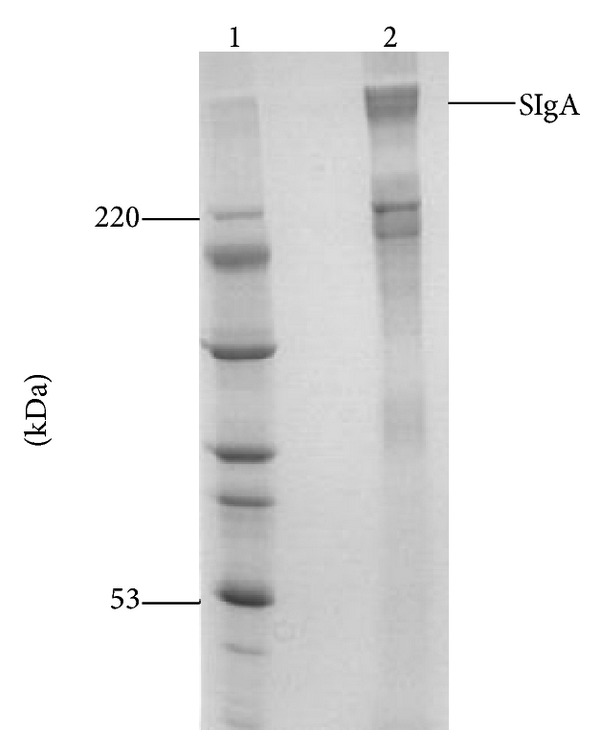
SDS-PAGE analysis of purified SIgA under non-reducing conditions. Lane 1: protein molecular weight marker; 2: purified SIgA antibody.

Recombinant SIgA was purified from cell culture supernatants, yielding approximately 25 mg of antibody per liter of supernatant. The final preparation was diluted in sterile PBS to 10 mg/mL. Using SDS-PAGE under non-reducing conditions; analysis of the purified antibody preparation demonstrated that the most abundant protein band was the purified SIgA, and the expected molecular size of the complex was 400 kDa (Figure 5). Other abundant protein bands were present at 200 kDa, which might be corresponding to monomers of IgA, or degraded products of the antibody preparation.

### 3.5. Neutralization Assay

**Figure 6:**
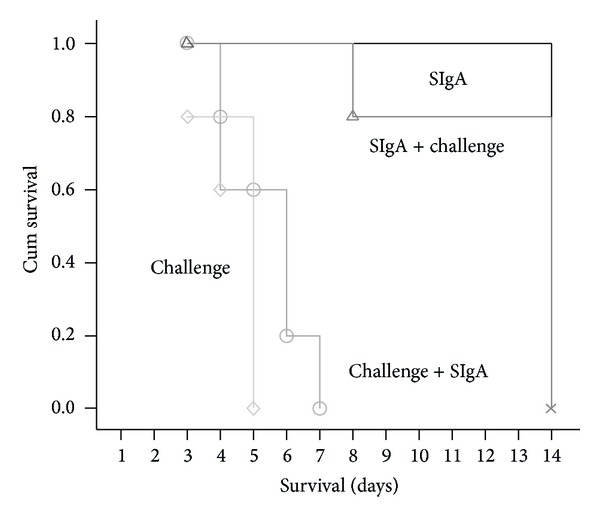
Survival analysis of BALB/c mice with different treatment. Kaplan-Meier survival analysis was used to determine the survival probability among the different groups. Mean survival days of the mice in Group 1 (SIgA + Challenge) was 12 ± 1 days versus 5 ± 1 days in Group 2 of Challenge + SIgA (*P* < 0.0001). *n* = 8.

The obvious CPE was observed around 48 h post-infection (p.i.). At 96 h p.i., CPE was evaluated and calculated for all wells. The TCID_50_ of the virus stock in MDCK cells was 10^−6.5^. When challenged with 100 TCID_50_ of virus, administration of 50 *μ*L of a 64-fold dilution of purified SIgA conferred complete protection at 96 h p.i. Therefore, the microneutralization titer of the SIgA solution was designated as 64. To examine the protective efficacy of the produced recombinant SIgA in vivo, we challenge the mice with a lethal dose of influenza A H5N1 A/Vietnam/1194/04 then administrated the mice with SIgA before or after challenging. When the mice in positive control group were infected with a dose of 10 LD_50_ of virus intranasally, they all died within 6 days p.i. In the negative control group, when administrated with SIgA alone, all the mice survived until sacrificed at 14th day. In SIgA + Challenge group, preadministration with SIgA provided a protection with survival rate of 80%. However, all mice in the group of Challenge + SIgA, in which the mice were administrated with SIgA after challenging, died at 7th day after infection. Kaplan-Meier analysis showed that pretreatment with SIgA could effectively prevent mice from lethal challenging (Figure 6) (*P* < 0.001).

## 4. Discussion

Antiviral drugs or vaccines are usually used to treat Influenza Virus infection. However, due to the prone mutation of Influenza Virus's genome, the virus easily acquires resistance to those available drugs. Using a vaccine to prevent AI would take 1-2 weeks to produce protective antibodies, during which time the virus could spread rapidly and cause severe health problems. So, developing a suitable agent for emergent prevention is prerequisite to control AI. The major invading routes of Avian Influenza Virus are the respiratory or digestive tract mucosa. Therefore, an agent targeting AIV on mucosa might bring effective protection in restricting AIV spreading, especially before the earliest stages of replication. In this study, we developed a chimeric SIgA as a passive agent to prevent the Avian Influenza H5N1 for the first time. A cell lines stably producing recombinant chimeric SIgA targeting AIV was constructed and the antiviral activity of the produced SIgA was evaluated in vitro and in vivo.

Production of SIgA normally requires the cooperation of two different cell types in the body. The heavy and light chains produced in plasma cells were assembled into IgA, which is on association with polymeric immunoglobulin receptor (pIgR) during transcytosing across the basolateral epithelial cells lining on the mucosa. The cytoplasmic and transmembrane region of the receptor was proteolytically cleaved, the truncated receptor termed secretory component (SC) and released from the receptor together with dimeric IgA. Previous experiments have shown that it is possible to assemble a functional SIgA molecule in vitro [22, 37, 39–41]. To clone the genes encoding SIgA subunits, highly specific and efficient primers (Table 1) were designed to amplify the exons directly from extracted genomic DNA. Sequence analysis confirmed that the cloned sequences were identical to the relative entries in the GenBank database. The “Genomic DNA Splicing” technique avoids RNA preparation and reverse transcription steps, and the entire assembly process can be finished within hours [33]. Because genomic DNA is more stable than RNA, it would be a more practical cloning strategy for many genes, especially for those that are very large where it is difficult to generate full-length cDNAs.

Heterologous protein expression is a complex interactive process between the vector and host cells. It is imperative to build a suitable vector to achieve the highest possible levels of protein expression. The eukaryotic expression vector used in this study, pEF-dhfr2a-NEO, was developed in our laboratory. This vector has many characteristics suitable for high-level expression of cloned DNA, including strong promoters, an SV40 late polyadenylation signal, introns, and two selectable markers. The vector has both CMV and human elongation factor 1 (EF1)-*α* promoter regions. The human CMV promoter is universally recognized as a strong promoter [38]. The EF1-*α* promoter (pEF) was cloned from the chromosome of human fibroblast cells [42]. EF1 is indispensable in protein synthesis and has a relatively high intracellular concentration. One study has shown that pEF is one of the strongest promoters discovered [43]. Polyadenylation has been shown to enhance RNA stability and translation [44]. Our vector contains an efficient SV40 late poly (A) signal downstream of the target gene [44–46], ensuring expression of foreign genes. Artificial chimeric introns can be used to reduce the chance of splicing and facilitate gene expression of cDNAs to a certain extent [47–49]. The vector pEF-dhfr2a-NEO includes an artificial intron in upstream of the target gene to avoid incorrect splicing and promote high levels of constitutive expression of the cloned DNA in mammalian cells. There are two selectable markers in pEF-dhfr2a-NEO, one of which is the dihydrofolate reductase (DHFR) gene [50]. The DHFR amplification system has become very popular for use in developing recombinant CHO cell lines. The DHFR system allows the gene of interest to be amplified many times by gradually increasing the concentration of MTX, an inhibitor of the DHFR enzyme. Utilizing this system can lead to increased levels of recombinant protein expression [51].

According to the patterns corresponding to H4L4JSC, H2L2, and HL complexes, the SIgA ratio in cell culture supernatants was much higher than that observed for dIgA and mIgA (Figure 4). The presence of bands that reacted with anti-*α*, anti-*κ*, anti-J, and anti-SC antibodies in high molecular weight species (H4L4J and H4L4JSC) confirms the covalent association of the light and J chains and the SC with heavy chains (Figure 4), which has been demonstrated previously [22, 37, 41]. These results indicated that we had successfully expressed the four polypeptides which efficiently assembled into entire SIgA antibody molecules in engineered CHO cells. The antigen-binding capacity of recombinant SIgA was measured by ELISA, and we demonstrated that SIgA had a strong capacity to bind antigens.

Recombinant SIgA was purified and the microneutralization assays were conducted by using the purified SIgA. When challenged with 100 TCID_50_ of virus, administration with 50 *μ*L of a 64-fold dilution of SIgA conferred complete protection at 96 h p.i. The microneutralization assay showed that SIgA had neutralization activity in vitro. On preadministrating with the purified SIgA, it can prevent 80% of the mice from lethal challenging; however, administration after infection could not bring effective protection to mice. It is not difficult to understand that preadministration would block virus entering the epithelial cells on mucosa. In contrast, when the virus finished the entry process, the SIgA locating on the surface of mucosa would not function to neutralize it. So we suggest that early administration is pivotal for SIgA functioning. Whether human components from the chimeric SIgA have effects on its potential efficacy was another important concern in designing chimeric antibody. The recombinant SC fragment used in this paper was of human origin, which had been proved to retain the function of directing SIgA distribution in the mucosa of mice [27]. Although the chimeric IgA exhibited the ability of binding to HA from Influenza virus H5N1 and neutralizing the virus in vitro, generally the chimeric antibody cannot retain one hundred percentage activity compared with its parental IgG molecule and this might influence its protective efficacy in mice to some extent. In conclusion, we have developed a recombinant anti-H5N1 SIgA in engineered CHO cells in this assay, which had a neutralizing capacity against influenza A H5N1 A/Vietnam/1194/04 in vitro and a protective capacity when administrated before challenging in vivo. Therefore, the developed SIgA in this research deserved to be further explored for application in emergent immunization.

## Figures and Tables

**Table 1:** Primers used in this assay.

Primer name	Primer sequence
mVK-f-ATG	ATGGAGWCAGACACACTCCT
mVK-r	GGATACAGTTGGTGCAGCATC
mVH-f-ATG	ATGGRATGGAGCTGGATCTT
mVH-r	ATAGACAGATGGGGGTGTCGTTTTGGC
IGA-mVHhCHf	TCTCCTCAGCATCCCCGACCAGCCCCAA
IGA-mVHhCHr	TCGGGGATGCTGAGGAGACGGTGACTGA
IGA-Hf-*EcoRI *	CGGAATTCACCACCATGGGATGGAGCTGGATCT
IGA-Hr-*Xbal *	GCTCTAGATCAGTAGCAGGTGCCGTCCA
IGA-mVKhCKf	AATCAAACGAACTGTGGCTGCACCATCT
IGA-mVKhCKr	CCACAGTTCGTTTGATTTCCAGCTTGGT
IGA-Kf-*EcoRI *	CGGAATTCACCACCATGGAGACAGACACACTCCT
IGA-Kr-*Xbal *	GCTCTAGACTAACACTCTCCCCTGTTGAAGCTCTTTGTGA
